# Modeling and De-Noising for Nondestructive Detection of Total Soluble Solid Content of Pomelo by Using Visible/Near Infrared Spectroscopy

**DOI:** 10.3390/foods12152966

**Published:** 2023-08-06

**Authors:** Sai Xu, Huazhong Lu, Xin Liang, Christopher Ference, Guangjun Qiu, Changxiang Fan

**Affiliations:** 1 Institute of Facility Agriculture of Guangdong Academy of Agricultural Sciences, Guangzhou 510640, China; xusai@gdaas.cn (S.X.); liangxin@gdaas.cn (X.L.); qiuguangjun@gdaas.cn (G.Q.); fanchangxiang@gdaas.cn (C.F.); 2 Guangdong Laboratory for Lingnan Modern Agriculture, Guangzhou 510640, China; 3 Guangdong Academy of Agricultural Sciences, Guangzhou 510640, China; 4 USDA, Agricultural Research Service, US Pacific Basin Agricultural Research Center, 64 Nowelo Street, Hilo, HI 96720, USA; chris.ference@usda.gov

**Keywords:** pomelo, total soluble solid content, nondestructive detection, modeling, de-noising, visible/near infrared spectroscopy

## Abstract

The flavor of Pomelo is highly variable and difficult to determine without peeling the fruit. The quality of pomelo flavor is due largely to the total soluble solid content (TSSC) in the fruit and there is a commercial need for a quick but nondestructive TSSC detection method for the industrial grading of pomelo. Due to the large size and thick mesocarp of pomelo, determining the internal quality of a pomelo fruit in a nondestructive manner is difficult, and the detection accuracy is further complicated by the noise typically generated by the common methods for the internal quality detection of other fruits. Thus, the aim of this study was to determine the optimal method to accurately detect pomelo TSSC and find a de-noising model which reduces the influence of noise on the optimal method’s results. After developing a full-transmission visible/near infrared (VIS/NIR) spectroscopy sampling method, the confirming experimental results showed that the optimal pomelo TSSC detection model was Savitzky Golay + standard normal variate + competitive adaptive reweighted sampling + partial least squares regression. The R^2^ and RMSE of the calibration set for pomelo TSSC detection were 0.8097 and 0.8508, respectively, and the R^2^ and RMSE of the validation set for pomelo TSSC detection were 0.8053 and 0.8888, respectively. Both reference and dark de-noising are important for pomelo internal quality detection and should be calibrated frequently to compensate for time drift. This study found that large sensor response translation noise can be reduced with an artificial horizontal shift. Data supplementation is efficient for improving the adaption of the detection model for batch differences in pomelo samples. Using this optimized de-noising model to compensate for time drift, sensor response translation, and batch differences, the developed detection method is capable of satisfying the requirements of the industry (TSSC detection R^2^ was equal or larger than 0.9, RMSE was less than 1). These results indicate that full-transmission VIS/NIR spectroscopy can be exploited to realize the nondestructive detection of pomelo TSSC on an industrial scale, and that the methodologies used in this study can be immediately implemented in real-world production.

## 1. Introduction

Pomelo (*Citrus maxima Merr.*) is a traditional Chinese fruit, with a cultivated area of more than 105,640 ha and an annual yield of 4,800,000 metric tons [1]. In addition, pomelo is also cultivated in other countries, such as Vietnam, Thailand, and South Africa, etc., and exported to Europe, Japan, and other regions [2,3,4]. While popular, the flavor of pomelo is quite variable. Pomelo flavor is largely based on the total soluble solid content (TSSC) in the fruit, which greatly affects the sweetness and can be difficult to determine without penetrating the peel [5]. Thus, there is a need within the pomelo industry to develop a quick, reliable, and nondestructive method for TSSC detection to provide quality control and uniformity in flavor for improved consumer satisfaction. The problem arises due to the thick mesocarp and large size of pomelo.

Traditionally, fruit TSSC is detected via using a refractometer on extracted juice, which is inefficient, wasteful, and ill-suited for large scale grading [6]. Zhang et al. found that the edibility of a pomelo could be nondestructively detected using X-rays [7]; however, the X-ray signal was largely unrelated to the TSSC. Our previous research results showed that the TSSC of pomelo can be roughly detected via external physical characteristics, which can be quickly acquired using machine vision technology; however, the coefficient of determination between the predicted TSSC value and the actual TSSC value was only 0.46 [8]. Our unpublished data showed that the pomelo TSSC was mostly unrelated to external electronic noise data. As a consequence, these pomelo TSSC detection methods were unsatisfactory.

Recently, visible/near infrared (VIS/NIR) spectroscopy technology has been widely applied for the nondestructive detection of TSSC in a variety of fruit, including apple [9,10], pear [11,12], peach [13], and orange [14]. The VIS/NIR spectrum absorbance is mainly affected by the stretched vibration overtones and combination modes of hydrogen-containing groups (X–H) including O–H, N–H, C–H, and S–H; thus, the TSSC can be calculated based on VIS/NIR absorbance data using an appropriate pattern recognition algorithm [15]. However, unlike other smaller fruits, the VIS/NIR signal, when transmitted through pomelo, is weak due to its large size, rough peel, and thick albedo, which result in a poor detection ability. In addition, the relatively lower signal noise ratio of VIS/NIR spectroscopy can further decrease the discrimination of this detection method. Thus, while VIS/NIR technology has the potential for use in nondestructive pomelo TSSC detection, both the modeling and de-noising must be improved to ensure its reliable application in industry.

Technically, there are three optical path structures for the internal quality detection of fruit using VIS/NIR spectroscopy, namely, reflectance spectroscopy [16], semi-transmission spectroscopy [17], and full-transmission spectroscopy [18]. Usually, reflectance spectroscopy is efficient for the internal quality detection of thin peel fruit, while transmitted spectroscopy is more efficient for the acquisition of internal information from fruit with a thicker peel. However, for the attainment of internal information from large-sized and thick-peeled fruit, semi-transmission spectroscopy is applied more often than full-transmission spectroscopy in order to improve the signal-to-noise ratio, as the optical length of semi-transmission spectroscopy is shorter than that of full-transmission spectroscopy [19]. Previous research has shown that TSSC can be detected using near-infrared hyperspectral imaging of a cut-open pomelo [20,21], that is, the internal quality of a pomelo can be inferred using NIR spectroscopy. Tian et al. showed that the internal TSSC of a pomelo could be detected using semi-transmission spectroscopy [5]. Semi-transmission spectroscopy has also been successfully applied for TSSC detection in watermelon [22]. However, while the in-variety variability of watermelon size and shape is low, the shapes of pomelo taper more and have a greater in-variety shape variability, making it difficult for an assembly line tray to adequately accommodate the shape of every pomelo in order for the VIS/NIR light source and receiving spectrometer to be properly aligned to transmit light fully through the pomelo. Thus, work to optimize the TSSC detection of pomelo based on full-transmission spectroscopy is still necessary for the industry. Our previous research results showed that the internal water content and degree of granulation of pomelos could be detected using full-transmission spectroscopy [23]; however, the water content of ripe pomelos is high and the differences in the degree of granulation result in obvious changes in histology, making the nondestructive detection of these attributes much less difficult than the nondestructive detection of the more subtle TSSC. Thus, further research is still needed to find an efficient nondestructive TSSC detection method for pomelo. 

Time drift, sensor response translation, and batch difference are three types of noise found in this study. Time drift noise is mainly due to the instability of the intensity of the light source as working hours increase [24], which changes the spectral sampling data, affecting the detection result. Reference and dark calibration have proven efficient for long-term time drift de-noising [25], but little research has focused on short-term time drift, which is more important in a low signal-to-noise ratio transmission, as is found with pomelo full-transmission spectroscopy. In our experience, sensor response translation noise occurs infrequently, and only occasionally occurred in this study. Sensor response translation mainly occurs due to inconsistency of the internal working of the spectrometer due to voltage fluctuations, which can result in a mismatch between the sensor response value and the wavelength. Thus, there is a requirement for a model with a solution for signal de-noising due to sensor response translation. Batch difference noise primarily occurs due to the background difference between practical detention samples (for model application) and modeling samples [26], which influences the detection result. Consequently, further research on de-noising is needed to ensure the stability of a pomelo TSSC detection model that is useful for industrial application. Previous research has shown that batch difference de-noising was beneficial for tomato quality detection [27]; however, whether batch difference de-noising can be beneficial for the low signal-to-noise ratio transmission obtained via pomelo full-transmission spectroscopy still requires further research.

Due to the large size and thick mesocarp of pomelo, determining the internal quality of a pomelo fruit in a nondestructive manner is difficult, and the detection accuracy is further complicated by the noise that is typically generated by the common methods for the internal quality detection of other fruits. To solve these issues, the main objective of this study was to use full-transmission VIS/NIR spectroscopy for the nondestructive detection of TSSC in pomelo fruit, and determine if this method, combined with an appropriate data de-noising and analysis model, was suitable for industrial application. The specific objectives of this study were to (a) develop an optimal method to accurately detect pomelo TSSC; (b) develop a de-noising model to decrease the influence of noise (time drift, sensor response translation, and batch difference) on pomelo TSSC detection data analysis.

## 2. Materials and Methods

### 2.1. Pomelo Samples

Pomelo (*Citrus maxima Merr.*), cultivar ‘honey pomelo’, harvested from Meizhou city, Guandong province, China were used for all experimentation. Harvest times, usage, sampling times, and sample sizes are shown in Table 1. There were 311 samples in total harvested for Batch 1 on August 10, 2022. From Batch 1, 132 samples were used at the first experimental hour for modeling research, while 60 were used at the second and third experimental hour each for time drift de-noising research. Fifty samples were used at the first experimental hour for sensor response translation de-noising research. There were 59 samples harvested in Batch 2 on August 30, 2022; All of which were used at the first experimental hour for batch difference de-noising investigations.

### 2.2. VIS/NIR Sampling Platform Set Up

**Figure 1:**
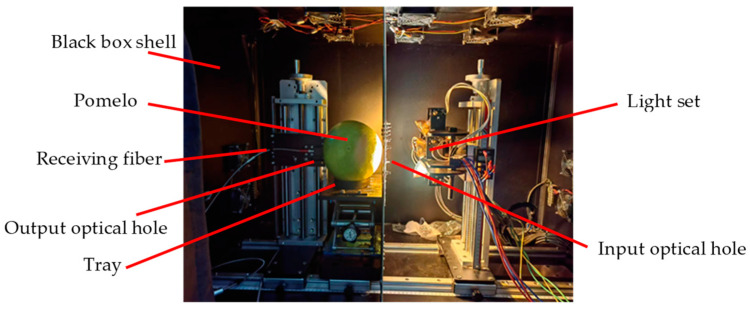
Structure of lab-developed VIS/NIR spectrum transmission sampling platform.

Our lab developed a full-transmission VIS/NIR spectrum transmission sampling platform, as shown in Figure 1. To mitigate extraneous light, pomelo samples were measured in a dark box. The 400 W (four 100 W halogen lamps) arc-shaped light set was on the right side. The lights were turned on 20 min before experimentation. In consideration of the practical needs of an assembly line detection, a movable tray was utilized to convey and stabilize each tested pomelo. The spectrum signal was transmitted through the pomelo from the right to the left side, was received by an optical fiber, and was then translated into a digital signal using a spectrometer (QE PRO with detectability for wavelengths between 400–1100 nm, Ocean Optics Inc., Dunedin, FL, USA). To avoid scattering noise being received by the optical fiber, all light went through both the input and output optical holes, passing through the pomelo fruit, before being detected by the optical fiber. The pre-sampling process was: (1) save the dark current value D, (2) save the reference value R (3.6 cm thick, spectral-calibrated panel made of barium sulfate material), and (3) finally, with the pomelo sampling detector response value (P), the pomelo transmissivity is equal to (P − D)/(R – D). After repeated adjustment, the optimal distance from the light set to the pomelo was set to 25 cm, and the optimal distance from pomelo sample to receiving fiber was set to 2 cm (a shorter distance can more efficiently avoid stray light). The diameter of the input and output optical holes was 7 cm and 1 cm, respectively, and the optimal integral time of spectrometer was set at 300 ms. Each sample was only sampled once to correspond to application on an assembly line.

### 2.3. TSSC Test

TSSC assessment was conducted subsequent to VIS/NIR spectrum acquisition by a digital pocket refractometer (PAL-BX/ACID1, ATAGO Co., Ltd., Tokyo, Japan). For TSSC assessment, pomelo samples were peeled to obtain fruit flesh, which was then crushed and homogenized, and the juice was filtered through gauze. Two drops of this juice were taken to directly measure TSSC. Each sample was measured three times, and the TSSC for that sample was recorded as the average of these three values. Between each measurement, the refractometer was calibrated with distilled water.

### 2.4. Modeling

**Figure 2:**
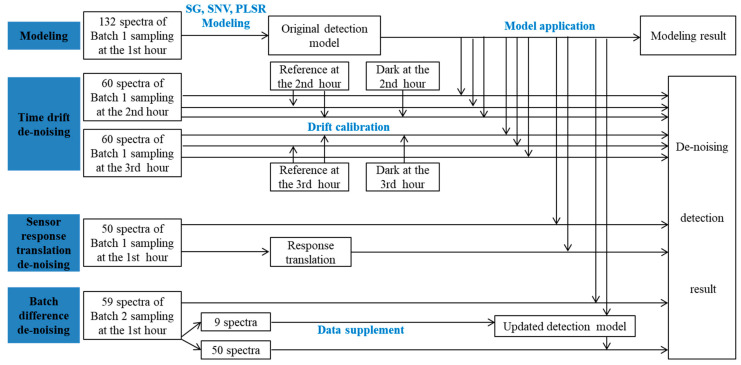
Workflow chart of the research process.

Savitzky Golay (SG) filtering [28], based on local least-squares fitting of data by polynomials, is a popular method for smoothing data. A SG filter was applied to reduce jitter noise (such as seen at 1000–1100 nm in Figure 2 of this study) due to the low signal-to-noise ratio of VIS/NIR spectrum transmission through pomelo. The effect of SG is influenced by the order of the polynomial and the size of the smoothing window. The standard normal variate (SNV) [29] method performs a normalization of the spectra that consists of subtracting each spectrum by its own mean and dividing it by its own standard deviation. SNV was applied to reduce the scattered noise, because, since there is space between the light source and the pomelo and between the pomelo and the receiving fiber, scattering noise is unavoidable. After applying SG and SNV for preprocessing, competitive adaptive reweighted sampling (CARS) [30] has the potential to select an optimal combination of the wavelengths existing in the full spectrum coupled with partial least squares regression by using the simple but effective principle of ‘survival of the fittest’, popularized by Darwin’s On the Origin of Species. CARS was applied for feature extraction among 939 spectroscopy response values (from 400 to 1100 nm). Partial least squares regression (PLSR) [31] is a technique that reduces the predictors to a smaller set of uncorrelated components and performs least squares regression on these components, instead of on the original data. As a fast, stable, and widely used method, PLSR was used on the data from the Batch 1 first-hour sampling group to investigate the modeling. One hundred pomelo samples were randomly selected as the calibration set, and the remaining 32 samples were used as the validation set. The matrix size used for PLSR modeling was feature number × sample number. For PLSR, the latent variables (LV) are the number of variables selected for model input after feature dimension reduction, which is the key parameter affecting detection accuracy, and which was determined by repeated testing in this study. The optimal LV number was selected at the calibration stage. The coefficient of determination (R^2^) is the key parameter for evaluating the correlation between the predicted value and the actual value. The range of R^2^ is from 0 to 1, where a greater R^2^ equals a better predictive ability (a stronger relationship between the predicted value and the actual value). Additionally, the root mean squared error (RMSE) is another way to evaluate a detection method; the closer the RSME value is to 0, the better the method’s prediction. Different combinations of the above methods were tested to compare modeling ability, namely, raw data + PLSR, raw data + SG + PLSR, raw data + SG + SNV + PLSR, and raw data + SG + SNV + CARS + PLSR. 

### 2.5. De-Noising for Model Application

To compare the effects of different time drift de-noising models, no de-noising, reference de-noising (spectrum calibrated by reference), and reference and dark de-noising (spectrum calibrated by both reference and dark) models were all applied. Reference de-noising transforms the sampling data utilizing the reference, where the sampling data are the full-transmitted spectrum of a pomelo, and the reference is the full-transmitted spectrum of a 2.5 cm-thick barium sulfate board. Reference de-noising transforms the sampling data to (data-dark)/(reference-dark), where dark is the response spectrum in a completely dark environment. For low signal-to-noise ratio data, significant time drift can affect the full-transmission spectrum of pomelo in a short period of term. Thus, time drift de-noising methods were performed on pomelo full-transmission spectrum data collected at the second and third hour (reference and dark were updated hourly). Sensor response translation de-noising was conducted by translating the sensor response back to the correct wavelength according to the dislocation distance, according to the length of translation. Batch difference de-noising was conducted by supplying new spectrum data from the new batch of pomelo samples to retrain the detection model to update the adaptive capacity of the detection model for further batch samples. All data analysis was performed using Matlab R2017a software (MathWorks Inc., Natick, MA, USA). The workflow of the modeling and de-noising research process is shown in Figure 2.

## 3. Results and Discussion

### 3.1. Modeling of TSS Detection

**Figure 3:**
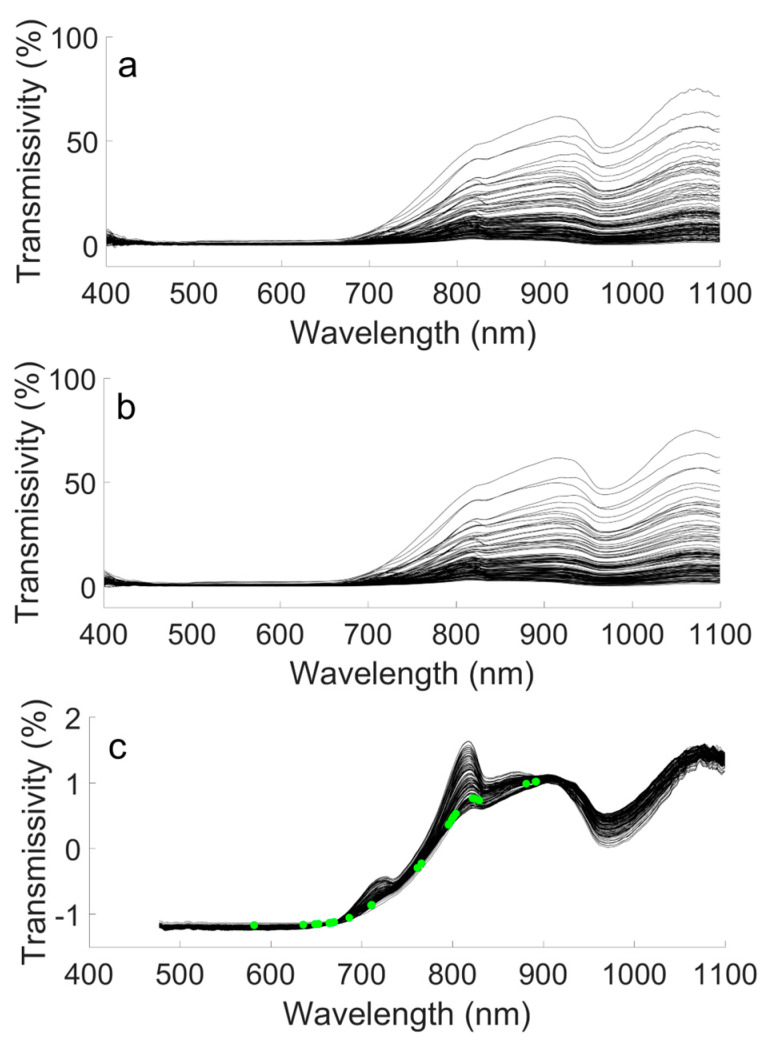
Spectrum of modeling pomelo samples: (**a**) raw spectrum; (**b**) SG-processed spectrum; (**c**) SG + SNV-processed and CARS feature-extracted spectrum.

The raw VIS/NIR spectrum transmitted through pomelo samples is shown in Figure 3a. The spectrum becomes irregular after 1000 nm; thus, third order 27-point SG processing was applied to eliminate the jitter noise, and the SG processing results are shown in Figure 3b. Information between 400 to 500 nm was removed because that wavelength area still contained significant jitter noise even after SG processing, and was therefore useless for TSSC detection. Finally, SNV was conducted to eliminate the scatter noise (the negative value is due to the SNV transformation of original data under the same standard), and 32 features (32 spectra, as the dots in Figure 3c) were extracted by CARS to reduce the redundancy of the input data of the detection model, with the results shown in Figure 3c. Previous research also found that the 600–900 nm range of the VIS/NIR spectrum was useful for the determination of the TSSC in other fruits [32,33]. The absorbance of VIS/NIR is mainly affected by the stretched vibration overtones and combination modes of color and hydrogen-containing groups (X–H), including O–H, N–H, C–H, and S–H [15]. Hence, the spectrum of pomelo is the superposition of the comprehensive response. For feature extraction, features with a stronger relationship with TSSC should be selected. However, highly related features might contain similar information. When this happens, a highly related feature combined with a feature with a lower relation may result in a better detection efficiency than combining two highly related features. Thus, feature selection is a complex combination issue. CARS provided an optimal feature combination, but it could not define the features which were unrelated to TSSC. 

The results of the different pre-processing methods combining PLSR modeling for pomelo TSSC detection are shown in Table 2. The results showed that SG, SNV, and CARS were all useful pre-processing models for pomelo TSSC detection, and that they all improved the detection accuracy. The optimal pomelo TSSC detection model was SG + SNV + CARS + PLSR, with an R^2^ and RMSEc of the calibration set for pomelo TSSC detection of 0.8097 and 0.8508, respectively, while the R^2^ and RMSEc of the validation set for pomelo TSSC detection were 0.8053 and 0.8888, respectively.

### 3.2. De-Noising of TSS Detection

#### 3.2.1. De-Noising of Time Drift

**Figure 4:**
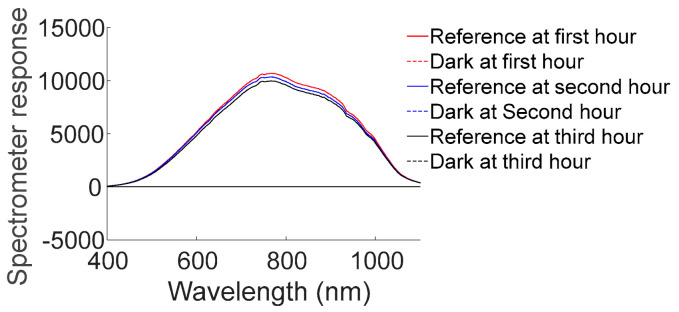
Spectrometer responses of reference and dark at different detection hours.

To visualize the effect of time drift on the spectrometer over a short period of time, the response of the reference and dark at the first, second, and third detection hour are shown in Figure 4. The spectrometer response value of the reference decreased with the increase in the working hours, but the spectrometer response value of the dark at different detection hours overlapped; that is, the spectrometer can work in a stable way in the short term, but the light source cannot. The reason for this is that the working process of a light source is an aging process, where the luminous flux of the light source attenuates with increases in the amount of time [34]. Thus, in practical application, the reference should be updated frequently to mitigate the noise due to time drift.

To further explore the influence of time drift noise on pomelo TSSC detection, the 132 samples from the first hour were used to investigate modeling based on SG + SNV + CARS + PLSR, while the 60 samples from the second hour and the 60 samples from the third hour were used to investigate the time drift de-noising effect using different methods (Table 3). Without de-noising, the detection accuracy of the second- and third-hour samples decreased when compared to the calibration accuracy of the first-hour samples. The R^2^ decreased from 0.8054 to under 0.5, and the RMSE increased from 0.8407 to more than 1.1. With reference de-noising, however, the detection accuracy of the second- and third-hour samples increased compared to those without de-noising, but the detection accuracy of the second-hour samples remained better than the third-hour samples. With reference and dark de-noising, the detection accuracy of the second- and third-hour samples was further improved, compared to only reference de-noising. We can infer that, when the spectrometer was in a completely dark environment, there was less effect to due to time drift than when measuring a full-transmission spectrum with a low signal-to-noise ratio. Thus, both reference and dark de-noising are important for pomelo internal quality detection, and spectrometers should be calibrated frequently to eliminate the effects that are due to time drift. Our previous research found that time drift noise has less influence on data acquisition for small-sized fruit, which can allow for the obtainment of a spectrum with a high signal-to-noise ratio [35].

#### 3.2.2. De-Noising of Sensor Response Translation

**Figure 5:**
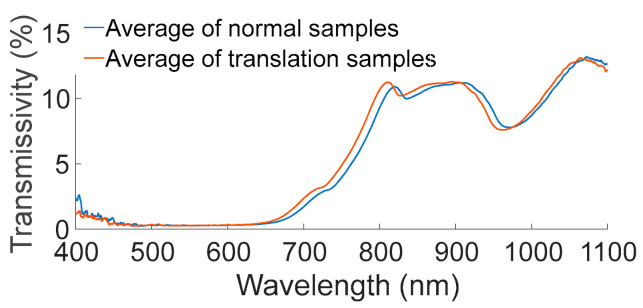
Average of normal samples and translation samples.

There were fifty samples for sensor response translation that were collected in the first detection hour, which is the first such report to the best of our knowledge. The averages of the 59 sensor response translation samples and the average of the 132 normal samples are shown in Figure 5. The curve shapes of the normal and translation samples were similar, but the response values of the translation samples were offset, being 8.9 nm lower. The reason for this may be an instability of the electronic components of the spectrometer. However, the exact reason requires further exploration.

To test if the sensor response translation noise could be reduced with an artificial horizontal shift, all of the response values of the translation samples were adjusted to be 8.9 nm higher. The TSSC detection results of the pomelo samples with and without de-noising are shown in Table 4. Due to the sensor response translation noise, the pomelo TSSC could not be efficiently detected with an R^2^ and RMSR of the validation set of 0.0872 and 2.1002, respectively. After de-noising, the R^2^ and RMSR of the validation set were improved to 0.6701 and 0.9277, respectively. Sensor response translation de-noising could not achieve detection results that were equal to the detection results from the samples without any interference from sensor response translation noise at all; however, the results were sufficiently close to satisfy industrial requirements (RMSE < 1). Usually, the deviation between the wavelength and spectrometer response is small [36] and does not affect the detection results. This study first found a large deviation (sensor response translation noise), and then a solution to provide as a reference for the industrial application of TSSC detection methods.

#### 3.2.3. De-Noising of Batch Difference

To test the adaptation of a pomelo TSSC detection model to batch differences, a detection model was built using the 132 Batch 1 samples from the first hour, and the 59 Batch 2 samples. The detection model could not adapt to different batch samples, and both the R^2^ and RMSR of the validation set were poor. Thus, 9 of the 59 Batch 2 samples were selected randomly to supply to the detection model to improve the adaptation to the new batch samples, and the remaining 50 samples were used for the validation set. After batch difference de-noising, the R^2^ and RMSR increased to 0.7038 and 0.8987, respectively. The results are shown in Table 5. Previous research has proven that data supplementation is efficient for improving detection model adaptation to batch differences in pineapple samples, where more supplemented samples resulted in an improved detection ability [37]. 

### 3.3. Comparison with Other Nondestructive Fruit Internal Quality Detection Research

Internal quality detection research based on VIS/NIR spectroscopy has mainly focused on small and thin-peel fruit, and less on pomelo due to its large size and thick peel. Previous research [22] has proven that pomelo TSSC can be nondestructively detected by semi-transmission spectroscopy. However, this method is difficult to fit into an assembly line due to the great in-variety shape variability of pomelo. The ability of full-transmission spectroscopy to carry out the nondestructive detection of pomelo TSSC still needs to be explored. Our previous research showed that water content and granulation can be nondestructively detected by full-transmission spectroscopy [26]. Thus, this study is an advancement of previous research. The research results proved that pomelo TSSC can be detected based on full-transmission spectroscopy.

SG, SNV, and CARS are commonly used preprocessing methods for spectrum data before modeling. For small fruit, these methods are not necessarily needed [38]. The application of these methods may cause signal distortion and overfitting of the modeling due to the signal-to-noise ratio being too high. Thus, combinations of these methods have often been tested to find the optimal preprocessing method [39]. This study proved that SG, SNV, and CARS are all efficient in pomelo VIS/NIR spectrum preprocessing, because the signal-to-noise ratio of the pomelo full-transmission spectrum is low.

Additionally, the stability of detection model applications has been less focused on, although this is especially important for the pomelo full-transmission spectrum with a low signal-to-noise ratio. For small-size or thin-peel fruit, time drift noise reduction (reference and dark calibration) was often conducted only once after starting up the detection equipment, as a small amount of drift would not affect the detection signal [40]. However, for the low signal-to-noise ratio sampling spectrum of pomelo, a small amount of drift in a short amount of time could affect the detection signal, and so time drift de-noising is better conducted hourly. Sensor response translation noise occurs infrequently and has not been reported in previous research on intelligent fruit quality detection, and only occasionally occurred in this study. This study was the first to find and provide a solution to sensor response translation noise for the industrial application of the detection model. Batch difference noise occurs not only in small-size fruit but also in large-size fruit, and is based on the growth characteristics, growth time, environment, and diversity of the specific agricultural product, and also affects the accuracy of the detection model. The data supplement provided in this study is suited for both small-size fruit [27] and large-size fruit.

These study results not only provide reference for the industrial application of the nondestructive detection of pomelo quality, but also provide reference for the stable model application of the nondestructive quality detection of other agro-products. A comparison of nondestructive internal quality detection of large- and small-size fruit is shown in Table 6.

## 4. Conclusions

This research was carried out to develop a nondestructive TSSC detection method for pomelo fruit based on full-transmission VIS/NIR spectroscopy for fast industrial on-line grading. Modeling and de-noising were of primary importance, due to the low signal-to-noise ratio of the transmission spectrum of pomelo. The experimental results indicated that the optimal pomelo TSSC detection model was SG + SNV + CARS + PLSR, with an R^2^ and RMSE of the calibration set for pomelo TSSC detection of 0.8097 and 0.8508, respectively, and an R^2^ and RMSE of the validation set for pomelo TSSC detection of 0.8053 and 0.8888, respectively. Both reference and dark de-noising are important for pomelo internal quality detection, and calibration should be performed frequently to eliminate the effects of time drift. This study was the first to find that a large amount of sensor response translation noise could be reduced via an artificial horizontal shift. Data supplementation was effective in improving the adaptation of the detection model with respect to batch differences in pomelo samples. With the de-noising model described above employed to reduce noise caused by time drift, sensor response translation, and batch difference, the detection ability of the model can satisfy the needs of the industry (TSSC detection R^2^ was close to or larger than 0.9, RMSE was less than 1). The results of this study verify that full-transmission VIS/NIR spectroscopy can be exploited to achieve the rapid nondestructive industrial-scale detection of pomelo TSSC, and that the major types of noise can be mitigated using appropriate model calibration, ultimately providing a fast and intelligent TSSC detection method and data de-noising and analysis model for the pomelo industry.

## Figures and Tables

**Table 1:** Experimental sample information.

Batch	Harvest Time	Usage	Sampling Time (hours)	Amount
1	August 10, 2022	modeling	1	132
	August 10, 2022	time drift de-noising	2	60
	August 10, 2022	time drift de-noising	3	60
	August 10, 2022	sensor response translation de-noising	1	50
2	August 25, 2022	batch difference de-noising	1	59

**Table 2:** Preprocessing and modeling results of pomelo TSSC based on PLSR.

Modeling Method	LVs	Calibration Set (The First Hour 100 Samples)		Validation Set (The First Hour 32 Samples)	
		R^2^c	RMSEc	R^2^v	RMSEv
Raw data+ PLSR	17	0.9344	0.4977	0.4989	1.4510
Raw data + SG + PLSR	17	0.7413	0.9792	0.4936	1.5788
Raw data + SG + SNV + PLSR	17	0.7638	0.8922	0.5715	1.4002
Raw data + SG + SNV + CARS + PLSR	22	0.8097	0.8508	0.8053	0.8888

**Table 3:** The second- and third-hour sample TSSC detection results based on the first-hour samples modeling under different de-noising methods.

	The 1st Hour Samples		The 2nd Hour Samples		The 3rd Hour Samples	
	R^2^	RMSE	R^2^	RMSE	R^2^	RMSE
Without de-noising	0.8054	0.8407	0.4862	1.1845	0.2578	1.5816
With reference de-noising	0.8054	0.8407	0.7538	0.8577	0.6881	0.8926
With reference and dark de-noising	0.8054	0.8407	0.7926	0.8450	0.7565	0.8531

**Table 4:** TSSC detection results with/without sensor response translation de-noising.

	Without De-Noising		With De-Noising	
	Calibration Set	Validation Set	Calibration Set	Validation Set
R^2^	0.8054	0.0872	0.8047	0.6701
RMSE	0.8407	2.1002	0.8413	0.9277

**Table 5:** TSSC detection results without/with batch difference de-noising.

	Without De-Noising		With De-Noising	
	Calibration Set	Validation Set	Calibration Set	Validation Set
R^2^	0.8054	0.5486	0.8032	0.7038
RMSE	0.8407	1.0715	0.8411	0.8987

**Table 6:** Comparison of nondestructive internal quality detection of large- and small-size fruit.

Fruit Size	Signal Noise Ratio	Preprocessing	De-Noising Method in This Study		
			Time Drift	Sensor Translation	Batch Difference
Small	High	Necessary	Suited, conducted only once after starting up	For reference	Suited
Large	Low	Unnecessary	Suited, conducted hourly	Suited	Suited

## Data Availability

The data used to support the findings of this study can be made available by the corresponding author upon request.
